# Breast cancer organoids and their applications for precision cancer immunotherapy

**DOI:** 10.1186/s12957-023-03231-2

**Published:** 2023-10-26

**Authors:** Dandan Guan, Xiaozhen Liu, Qingyang Shi, Bangjie He, Chaopeng Zheng, Xuli Meng

**Affiliations:** 1 College of Medicine, Soochow University, Soochow, China; 2 General Surgery, Department of Breast Surgery, Cancer Center, Zhejiang Provincial People’s Hospital, Hangzhou Medical College, Hangzhou, 310014 Zhejiang China; 3 Key Laboratory for Diagnosis and Treatment of Upper Limb Edema of Breast Cancer, Hangzhou, Zhejiang China; 4 Key Laboratory for Diagnosis and Treatment of Endocrine Gland Diseases of Zhejiang Province, Hangzhou, Zhejiang China; 5 Department of Urology, Haining Central Hospital, Haining Branch of Zhejiang Provincial People’s Hospital, Jiaxing, Zhejiang China; 6 Department of General Surgery, Traditional Chinese Medicine Hospital of Zhuji, Zhuji, Zhejiang China; 7 Zhejiang Chinese Medical University, Hangzhou, Zhejiang China

**Keywords:** Breast cancer, Immunotherapy, Organoid, Tumor microenvironment

## Abstract

Immunotherapy is garnering increasing attention as a therapeutic strategy for breast cancer (BC); however, the application of precise immunotherapy in BC has not been fully studied. Further studies on BC immunotherapy have a growing demand for preclinical models that reliably recapitulate the composition and function of the tumor microenvironment (TME) of BC. However, the classic two-dimensional in vitro and animal in vivo models inadequately recapitulate the intricate TME of the original tumor. Organoid models which allow the regular culture of primitive human tumor tissue are increasingly reported that they can incorporate immune components. Therefore, organoid platforms can be used to replicate the BC–TME to achieve the immunotherapeutic reaction modeling and facilitate relevant preclinical trial. In this study, we have investigated different organoid culture methods for BC–TME modeling and their applications for precision immunotherapy in BC.

## Background

Breast cancer (BC) has the highest incidence rate  worldwide and the highest mortality rate among cancers in women [1, 2]. Treatments of BC mainly include surgery, radiotherapy, endocrine therapy, systemic chemotherapy, and antihuman epidermal growth factor receptor 2 (HER2)-targeted therapy. Individualized precision therapy is usually tailored to the clinicopathological and molecular characteristics of patients with BC. Despite significant advances in BC treatment, proximately 20% of BC patients may still relapse or metastasis relapse or metastasis, and treating them is still a challenge [3].

Owing to the recent rapid developments, immunotherapy has gradually become an efficient treatment for cancers [4], which targets the intrinsic immunity of the patients [5]. The immunotherapeutic approaches include oncolytic viruses, immune checkpoint blockade (ICB) therapy, pattern recognition receptor-targeted therapies, adoptive cell transfer (ACT), and adjuvants [6–8]. However, only a subset of patients with specific tumor types benefits from immunotherapy.

Because of the low infiltration of lymphocytes in breast tumors, BC was formerly deemed immunologically “cold” [9]. However, increasing evidence has indicated the prominent heterogeneity of BC regarding the tumor microenvironment (TME) and immune infiltration [10, 11]. Immunotherapeutic approaches in conjunction with classic treatments have been explored for maximizing anti-BC efficacy, especially in triple-negative breast cancer (TNBC). A large-scale clinical trial has shown that the combination of chemotherapy and pembrolizumab, a kind of programmed cell death 1 (PD-1) suppressants, could help in achieving significantly and clinically meaningful benefits in both disease-free survival and overall survival in programmed death ligand 1 (PD-L1)-positive ([combined positive score≥10) patients with advanced TNBC [12, 13]. Nowadays, this combination therapy has become a recommended first-line treatment for advanced patients with PD-L1-positive TNBC. Furthermore, in patients with early TNBC, the addition of pembrolizumab to neoadjuvant chemotherapy, followed by adjuvant pembrolizumab after surgery, contributed to a significantly higher pathological complete response and longer event-free survival [14]. Nevertheless, the research on the value and further application of immunotherapy in BC is far from enough.

The role of TME in diverse aspects of tumor development, such as vascularization, immunity, and tissue metabolism, has been demonstrated and is well-acknowledged [15–17]. TME consists of carcinoma cells, extracellular matrix (ECM), stromal cells (e.g., vascular endothelial cells, myoepithelial cells, and fibroblasts), and immunocytes (e.g., B cells, T cells, natural killer [NK] cells, and macrophages). Immunotherapies facilitate the systemic immunologic monitoring and locally modulate the tumor immune microenvironment (TIME) [18]. BC–TME has crucial clinical significance in patients with BC [19]. For further research and application of immunotherapies in BC, it is imperative to reconstruct the BC–TME and investigate the cell interplays in BC–TME, which are environment-dependent intricate processes. Appropriate preclinical methods should be established that can reliably recapitulate the composition and functions of BC–TME. From cell co-cultures to different animal models, there are various models for immunotherapeutic research; however, they cannot completely recapitulate the intricate TIME of patients with BC at present. Nevertheless, the novel organoid models can simulate immunotherapy response and promote immunotherapy research. In this study, we summarize the common immune organoid models of BC and introduce their applications.

## Conventional models for immunotherapy

Two-dimensional (2D) in vitro models are predominant preclinical models for various types of studies because they are cost-efficient, relatively simple, and adaptable to toxicity research and high-throughput screening [20]. However, 2D models are not suitable for immunological research due to the following reasons: 2D models are often cellular monocultures and unable to recapitulate the entire fundamental cellular compositions and cell interplays in vivo, especially the interplays between ECM and immune cells [21–23]; the carcinoma-derived cells may acquire substantial genetic alterations and are unable to represent TME and tumor heterogeneity of the native tumor tissue [23, 24].

In vivo models are beneficial for the toxicology and efficacy research of classic drugs. But they cannot assess all types of immunotherapies, owing to the huge inherent disparities in immune systems between animals and humans [25]. Patient-derived tumor xenograft (PDTX) models can partially recapitulate the cancer cell interplays with the stromal cells and ECM and partial interplays with the immune response [23] and are already used for biomarker identification, preclinical drug testing, cancer research, and drug discovery [8, 26]. Nonetheless, PDTX models still lack key immunity components of humans, such as circulating B and T cells. To solve this problem, humanized models of immuno-oncology are established by transplanting tumor fragments obtained from patients into the human immunocyte-bearing mouse model. However, the establishments of these models are fraught with challenges, considering the cost, yield, time, and complete immune compatibility [6, 7].

## Organoid technology for immunotherapy

### Overview of current BC organoid (BCO)

Three-dimensional (3D) multicellular architectures, such as organoids, can mimic the original tissue after being cultured in a 3D matrix [27, 28]. Hans Clevers' team [29] successfully cultured mouse intestinal organoids in vitro, which started the prelude of organoid technology. After more than a decade of development, organoids have gradually become a new in vitro model for biomedical research and a powerful tool to maintain the characteristics of original cells in a near-native state. Patient-derived organoids (PDOs) are 3D models cultured in a 3D matrix, which derived from original patient tumors. PDO models closely mimic in vivo conditions of the original tumors, allowing for the in vitro modeling of cancer and personalized tumor response testing [30]. To date, PDO biobanks have been created from various carcinomas, including that of the breast [31], prostate [32], ovary [33], lung [34], stomach [35, 36], colorectum [37–40], liver [41], pancreas [42], and brain [43]. Robust BCO models have been established since 2018 and have been shown to replicate the original breast tumors satisfactorily, regarding statuses of HER2, hormone receptor (HR), and morphology, thus enabling in vitro drug screening [31]. During the culture of BCOs, the culture medium is added with various growth factors and/or pathway suppressants based on the type of tumor [44–46]. More efficient approaches to culture and characterize BCOs have been developed, and various clinical BCs have been duplicated by BCO models. Nevertheless, in earlier studies, the BCO models with immune components which were suitable for immune research had not been established.

**Fig. 1:**
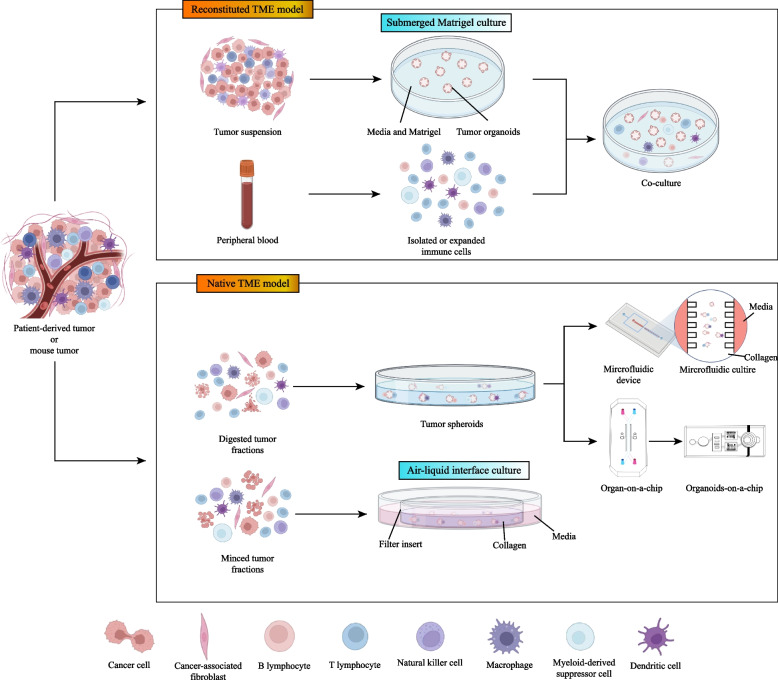
The major approaches for modeling the immune-breast cancer organoids (BCOs). In reconstituted approach, BCOs are cultured in extracellular matrix (e.g., Matrigel) and submerged beneath tissue culture medium. Exogenous immune cells, such as stromal cells or additional immunocytes, are isolated and co-cultured with BCOs. In native tumor microenvironment (TME) models, the intrinsic immune microenvironment of tumor tissues is preserved without reconstruction. Tumor spheroids from digested tumor fractions can be mixed with collagen and added into organ-on-chips or microfluidic devices. In air-liquid interface (ALI) culture, minced tumor fragments containing both tumor cells and immune cells are embedded in collagen gels within an inner Transwell dish

For many years, tremendous efforts have been devoted to exploring novel approaches for the co-culture of immune cells and organoids in various cancers, including BC. The immune organoid models suitable for immunotherapy include reconstructed TME models and native TME models (Fig. 1). In reconstructed TME models, such as submerged Matrigel culture, isolated or expanded immune cells are added to the submerged Matrigel culture systems of BCOs. In native TME model strategies, including microfluidic 3D culture and air–liquid interface (ALI), the small tumor tissue fragments and native TME are retained as a holistic unit without artificial reconstitution [18]. Herein, we discussed the diverse co-culture approaches for recapitulating BC–TME and the applications of sophisticated BCOs for precise cancer immunotherapy.

### Various culture strategies for BCOs

#### Retaining and expanding endogenous immune cells within PDOs

One approach for native TME models is retaining and expanding endogenous immunocytes within organoids as a cohesive unit, which is a relatively simple immunotherapeutic approach. This co-culture model can be applied in the study of breast. Zumwalde et al. [47] successfully established organoids sourced from the mammary ductal epithelial cells of the human. Apart from specifying the intraepithelial lymphocyte compartment of healthy human breast tissues, they also pinpointed a T-lymphocyte subset, whose BC cell reactivity could be increased through pharmacological targeting. According to their findings, the leukocyte communities of breast organoids were unlike the peripheral blood counterparts, and the utilization of Vδ2 (+) T-cell reactivity to the Food and Drug Administration-approved bisphosphonates, as a novel immunotherapeutic strategy, could suppress BC growth. This is the first immunocyte-organoid co-culture model reported in the immunological study of BC.

#### Submerged Matrigel culture

The submerged Matrigel system is extensively used for culturing patient-derived carcinoma cells in a mixture of tissue culture medium and 3D matrix Matrigel. But stromal components are not retained by the regularly submerged Matrigel organoids. For the precision study of BC–TME and immunotherapy, ECM, stromal cells, and additional exogenous immunocytes are required to reconstruct BC–TME. Many studies have reported their novel models for co-culturing BCOs and immune cells. Hanley et al. [48] developed BCO-autologous stromal cell co-culture systems and revealed their dynamic molecular interactions. They further demonstrated that the infiltrative capacity and molecular phenotype of BC cells could be affected by the adjacent mammary cancer-associated fibroblasts (CAFs). Dhimolea et al. [49] co-cultured HR-positive BC cell line spheroids or PDOs in 3D ECM, alone or together with bone marrow stromal cells (BMSC), and highlighted the role of BMSC in affecting metastatic microenvironment and mediating hormone-independent tumor growth. These studies showed that adding additional immune cells and other stromal cells to BCOs cultured by submerged Matrigel culture is a feasible approach to reconstruct BC–TME. However, matrix components and immune cells are usually not fully preserved in tissue processing stage, and restoring BC–TME to its initial stage is a laborious and time-consuming process. This is also a common problem in other co-culture approaches, such as ALI culture and microfluidics 3D culture. Solving this problem is of great significance to the development of immune organoid models.

#### ALI culture

Through the ALI method for creating organoids, the tumors can grow as a cohesive unit, and en bloc preservation of carcinoma cells can be allowed, retaining their native stroma. Tumor organoids developed from minced fragments of primary tissues are added to a collagen gel that is inside an inner Transwell dish. Culture medium diffuses into the inner dish from the outer dish via the permeable Transwell. The ALI method is applied to expose the top collagen layer to the air, so the cells receive adequate oxygen [50, 51]. This type of organoid culture achieves the TME recapitulation by intrinsically retaining the fibroblasts and multiple parental tumor immunocytes, such as various immune cells (B cells, T cells, NK cells, and macrophages), without requiring artificial reconstruction, making them distinct from submerged Matrigel culture.

Presently, it has been observed that a PD-1 blocking antibody can initiate anti-tumor immune responses within ALI organoids from several types of carcinomas, in an approach that seems independent the PD-L1 expression status of tumors [52]. Notably, the ALI culture fails to remedy the short preservation deficiency of stromal myofibroblasts, such as SMA and vimentin, which would decline over a 6-week period in the organoid cultures [53]. It is challenging to investigate innovative approaches to ALI culture for breast organoids. The ALI-PDOs en bloc having endogenous immune stroma is less effective in breast organoids, because growing BCOs and maintaining TILs derived from BC are incredibly more challenging than the rest of the carcinoma types. Preserving the vascular system that carries immune cells may be helpful for ALI culture, but perfusion remains a huge challenge.

#### Microfluidics 3D culture

A collagen gel mixture can be used to culture murine- or patient-derived organotypic tumor spheroids (MDOTS/PDOTS) in microfluidic 3D devices [54]. The patient-derived samples of tumor tissues were subjected to enzymatic and mechanical fragmentation for the MDOTS/PDOTS culture. The obtained samples were a nonuniform mixture of macroscopic tumor fragments, single cells, and spheroids [55]. Tumor spheroids are grown in a media-assisted 3D gel in the central zone of the media channels of the microfluidic 3D device, which runs parallel and is situated on either side of the central zone. Cultivation and assessment of MDOTS/PDOTS from syngeneic immunoreactive murine models and tumor samples of patients are achieved for 1–2 weeks, such as Merkel cell carcinoma and melanoma [56, 57]. As shown by flow cytometric profiling of immunocytes, apart from retaining tumor cells, the MDOTS and PDOTS also retain myeloid populations (tumor-associated macrophages, monocytes) and autologous lymphocytes (B and T cells). The results showed that these models can retain myeloid cell and autologous lymphoid communities and respond to immune checkpoint blockade (ICB) in 3D short-term microfluidic culture. Therefore, MDOTS/PDOTS profiling is an innovative platform for ICB assessment, where the clinically relevant specimens of patients and recognized murine models are utilized. Through this in vitro culture, a preclinical model of BC was also developed [58]. This model has certain value in screening the classic chemotherapeutic agents in real time and great potential to play a role in immune studies. Furthermore, Truong et al. [59] co-cultured BC cells and patient-derived fibroblasts in 3D tumor and stromal sites so that the TME spatial organization can be mimicked on a microfluidic chip. They studied the tumor-stroma interactions and further revealed that CAFs promoted invasion through the upregulation of glycoprotein nonmetastatic B in BC cells. These outcomes indicated the ability of this co-culture model to recapitulate patient-specific TME for exploring the tumor–stroma interactions in BC. Therefore, microfluidics 3D culture is a promising tool to co-culture organoids and immune cells for immunological research on BC. In terms of limitations, specialized equipment is required, and the immune components may decline over time in microfluidics 3D culture.

#### Organoid-on-a-chip

Organ-on-chip (OoC) is an emerging technology combining cell biology, microfabrication, and microfluidics. Epithelial organoid cultures can be integrated into organ-on-a-chip platforms to form the “organoid-on-a-chip” system, a more complex culture system for organoids [60, 61]. “ Organoid-on-a-chip” is a biochip system that combines the technological advantages of organoids and organ-on-chip and produces the same physiological and metabolic linkage reaction with multiple human organs. This model can overcome the disadvantages of organoids by making organoids more uniform and mimicking the bodily physical conditions, e.g., by providing culture media perfusion. Besides, with the aid of integrated sensors and actuators, microfluidic devices can be used to perform parameter assay as well as culture condition surveillance and control [62]. The multi-organoid-on-a-chip was also established [62, 63]. Critical parameters of the immune microenvironment and TME are recapitulated via the organoid-on-a-chip platform of tumors so that the synergistic and independent effects of various tumor progression components can be systematically comprehended [64, 65]. This co-culture model has progressively become an innovative and reliable tool for investigating how tumors evade immunity by affecting TME and how they resist immunotherapy.

In the field of BC research, organoid-on-a-chip has been used for surveilling the primary tumor responses to immunotherapies in patients. Zhang et al. [66] reported that MCF7 and MDA-MB-231 BC cells have been used to investigate whether on-chip testing of personalized immunotherapy was achievable by applying their mini-tumor chip. Herein, primary tumors were loaded on a chip after dissociation into single cells. On this basis, the primary BC cells on-chip were capable of responding to the anti-PD1 therapy or NK cell therapy at varying efficiencies. This on-chip reaction stresses the effectiveness of the chips in assessing the immunotherapy responses of patient tumors, with great potential to become the gold standard for preclinical screening of individualized therapy. However, the improvement of mechanical conditions and culture media for varying tissues is still tremendously challenging. Besides, whether current on-chip tumor models can fully simulate biological processes in vivo remains unclear, their application value should be investigated for individualized immunotherapy by clinical results in the future.

### The applications of organoids for immunotherapies

An ideal preclinical platform for immunotherapy screening and research requires a co-culture model of cancer cells and immune cells, which completely reflects the heterogeneity of the original TME. Recent progresses concerning sophisticated tumor organoids have suggested that BCOs can be regarded as ideal models for evaluating immunotherapy efficacy and identifying innovative combinatory therapy strategies. The immunotherapy applications of the organoids technology in BC are discussed below (Table 1).

**Table 1:** Organoids models and their application for immune researches in BC

**Authors**	**Years**	**BC cells**	**Co-culture cells**	**Approaches**	**Application**
Hanley, C. J. et al. [48]	2020	Primary BC cells	ECM	Adding stroma cells to organdids	Reveal dynamic molecular interactions between stromal cells and cancer cells
Dhimolea, E. et al. [49]	2021	HR+ BC cells	ECM +/− BMSC	Adding cells to organdids	Characterize the pleiotropic hormone-independent mechanisms in HR+ BC tumors
Zumwalde et al. [47]	2016	Human breast ductal epithelial cells	Vδ2+ T lymphocytes	Retaining and expanding endogenous immune cells	Demonstrate the ability of Vδ2(+) T cells to respond to bisphosphonate drugs as an immunotherapy in BC
Aboulkheyr Es, H. et al. [58]	2022	PDOTS	Original TME and infiltrated immune cells	3D microfluidic device	The real-time screening of conventional chemotherapy agents
Truong, D. D. et al. [59]	2019	BC cells	CAFs	3D Microfluidic Device	Demonstrate the ability of this co-culture model to recapitulate the TME
Zhang et al. [66]	2022	MDA-MB-231 cells	CAFs, ECMs, HMFs	Organ-on-chip	Monitor patient primary tumors’ responses to anti-PD1 treatment
Wallstabe, L. et al. [67]	2019	MDA-MB-231	ECM	Microphysiologic 3D culture	ROR1-CAR T-cell therapy
Thakur, A, et al. [68]	2021	Multiple BC cell lines	PBMC or MDSC	3D tumorsphere model	Adoptive transfer of bispecific antibody armed activated T cells
Ayuso et al. [69]	2019	MCF7 cells	ECM	3D microfluidic device	NK cells immunotherapy
Dees, S. et al. [70]	2021	TNBC cells	Human PBMCs	Adding cells to organoids	Combining T-cell-redirecting bispecific antibodies with ICIs
Shelkey, E. et al. [71]	2022	4T1 TNBC murine cells	Matched splenocytes	Adding cells to organoids	Investigate the factors affecting ICB response
Zhou, Z. et al. [72]	2021	MDA-MB-468 cells	CAFs, CD8+ T cells	Adding cells to organoids	A high-throughput screen to identify epigenetic inhibitors
Carter, M. E. et al. [73]	2022	BC tumor tissues	_	_	Investigate the effects of oncolytic virotherapy
Behrens, M. D. et al. [74]	2022	MDA-MB-231, BC PDX	_	_	Investigate the effects of oncolytic virotherapy

Notes: *BC* breast cancer, *HR* hormone receptor, *CAFs* cancer-associated fibroblasts, *3D* three dimensions, *ECM* extracellular matrix, *NK* natural killer, *BMSC* bone marrow stromal cells, *PDOTS* patient-derived organotypic tumor spheroids, *TME* tumor microenvironment, *HMFs* human mammary fibroblasts, *PD-1* programmed cell death protein 1, *CAR-T cell* chimeric antigen receptor T cell, *ICIs* immune checkpoint inhibitors, *PBMCs* peripheral blood mononuclear cells, *MDSC* myeloid-derived suppressor cell, *ICB* immune checkpoint blockers, *PDX* patient-derived tumor xenograft

#### ACT

Cellular immunotherapy, also known as ACT, harnesses the killing power of immune cells to fight against cancer. The initial step of ACT immunotherapy is the isolation of immunocytes from either a patient (autologous cells) or a donor (allogeneic cells), which are subsequently genetically engineered, expanded, and activated ex vivo and eventually reinjected into the patients [75, 76]. Recently, ACT has made significant research and clinical advances in many types of cancer. The major ACT therapies include engineered T-cell receptor (TCR), NK cell, tumor-infiltrating lymphocyte (TIL) [77], and chimeric antigen receptor (CAR) T-cell therapies.

As mentioned above, Zumwalde et al. [47] successfully co-cultured Vδ2+ T lymphocytes with organoids derived from human breast mammary ductal epithelial cells and demonstrated the potential of utilizing Vδ2 (+) T cells to respond to bisphosphonate drugs as the novel immunotherapy approach to inhibit BC growth. This study first utilized organoid models for ACT therapy.

CAR-T-cell therapy refers to one type of ACT immunotherapies. CAR-T cells are equipped with specific antibodies to recognize antigens in autologous tumor cells and further induce cytotoxic effects, achieving remarkable successes in the treatment of hematologic malignancies recently. CAR-T-cell therapy also exerts a role in the treatment of BC but is not as effective as expected [78]. The main reason is probably that solid tumors usually face multiple barriers to ACT, such as immunosuppressive TME, antigen specificity, and toxicities. BCOs may be efficient platforms for showing TME and assess the tumor-specific cytotoxicity of T cells. Wallstabe et al. [67] once established standardized and scalable BCOs from MDA-MB-231 with architectural and phenotypical features of TNBC. Using these 3D tumor models, they investigated the antitumor function of CAR-T cells and obtained proof of concept for their safety and efficacy before the clinical application. They further demonstrated potent antitumor effects of receptor tyrosine kinase-like orphan receptor 1-specific CAR-T cells.

Adoptive transfer of bispecific antibody-armed activated T cells (BATs) exhibited promising antitumor activity in the clinical trials of solid tumors. Thakur et al. [68] hypothesized that the release of BAT-induced tumor-targeting effectors (TITE) might play the role of a potent antitumor and immune-activating immunotherapy. The TITE exhibited potent cytotoxic activity against multiple BC cells in a 3D tumorsphere model. They believed that TITE could offer a clinically controllable cell-free platform to target various tumor types containing BC, regardless of the mutation-prone and heterogeneous nature of the tumor cells.

The efficacy of NK cell immunotherapy in BC was also evaluated by a microfluidic platform [69]. This model contains a 3D BC spheroid in a 3D ECM and two flanking lumens lined with endothelial cells, replicating pivotal structures and components within the immune response. It was discovered that NK cells could detect tumor spheres farther and faster than antibodies. Once inside the spheroid, NK cells can also destroy tumor cells completely, both at the spheroid periphery and the innermost layers. Besides, Yang et al. [79] showed that mesothelin-targeted CAR-NK cells derived from induced pluripotent stem cells had a certain efficacy in killing TNBC cells in several preclinical models, including in vitro organoid models. Parikh et al. [80] established PDOs from common epithelial cancers, including BC, and demonstrated their utility as an effective tool for selecting TCRs and TIL in ACT.

All the obtained findings showed the application of the model for detecting novel therapeutic approaches of ACT to increase immunotherapy against solid tumors.

#### Antibody-based immunotherapy

Antibody-based immunotherapy is a leading type of cancer immunotherapy that particularly and directly restricts cancer cell survival, activates the immune system to eradicate cancer cells, or delivers cytotoxic compounds [81]. Recently, tumor organoids have been used as preclinical models to investigate the efficacy of antibody-based checkpoint blockade immunotherapy. The immune–tumor organoids are also used for detecting new strategies for an antibody-based combination treatment of cancers.

Organoid models have been used to research the value of antibody-based therapeutics in TNBC. Dees et al. [70] reported that the treatment of trophoblast cell-surface antigen 2 (Trop2) and trophoblast cell-surface antigen 2 (CEACAM5) expressing 3D-TNBC spheroids with CD3 × Trop2 or CD3 × CEACAM5 bispecific antibodies in combination with human peripheral blood mononuclear cells significantly hindered the TNBC cell growth. Besides, the addition of an antagonistic anti-PD-1 monoclonal antibody to this model further increased cell death in 3D TNBC spheroids. These findings indicated that combining T-cell-redirecting bispecific antibodies with immune checkpoint inhibitors (ICIs) provides a practical approach to improving antitumor efficacy and surmounting the immunosuppressive TME in TNBC. Ning et al. [82] reviewed representative cutting-edge antibody-based therapeutics in TNBC in clinical use and trials and suggested that antibody-based therapeutics hold great promise in TNBC. For a better clinical application of antibody-based therapeutics in TNBC, effective preclinical verification using reliable models is particularly crucial. Considering the advantages of immune–tumor organoids in reconstructing the TME, BC organoids can be an ideal model for future studies on antibody-based therapeutics in BC.

#### ICB therapy and combination therapy

ICB therapy has exhibited promising results in various cancers. In recent years, extensive efforts have been made to develop effective immunotherapy to enhance clinical outcomes in BC, particularly for TNBC. Growing evidence suggests that BC is markedly heterogeneous concerning immune infiltration and the TME, and that lymphocyte infiltration into tumors is related to a better prognosis and better clinical responses to chemotherapy [83, 84]. However, genetic heterogeneity, the lack of actionable targets, and immune evasion lead to limited clinical response rates to ICB therapy. In metastatic BC, lasting responses occur in only approximately 5% of patients and are mostly limited to TNBC [85]. When ICB is used in combination with chemotherapy in patients with PD-L1-positive tumors, response rates may increase. But most BC patients do not benefit from ICBs. To further study the response of BC to ICB, better immune models in vitro on antitumor immunity are urgently needed.

A study previously showed that PDOs could functionally recapitulate the PD-1/PD-L1-dependent immune checkpoint system, thereby allowing the in vitro modeling of intrinsic and syngeneic immune cell responses [86]. Shelkey et al. [71] proposed a novel immune-enhanced tumor organoid (iTO) system to explore factors affecting the response to ICB therapy. They successfully showed the response to ICB therapy using the 4T1 TNBC murine cell line and matched splenocytes. Furthermore, the administration of bacterium derived from species found in the immunomodulatory host microbiome could increase the ICB-induced apoptosis of tumor cells and decrease the levels of the immune cell receptor. These results showed an approach to isolate individual factors that altered the response to ICB and streamlined the study of the effects of the microbiome on ICB efficacy. On the basis of these results, we can conclude that iTOs are robust platforms that help assess the efficacy of cancer immunotherapy, discover immune-oncology resistance mechanisms, and identify new combination therapies for ICB therapy. Zhou et al. [72] reported a high-throughput immune-drug screening approach on the basis of the functional interaction of mouse or BCOs and tumor-specific cytotoxic T cells. On this basis, they identified that the epigenetic inhibitors GSK-LSD1, CUDC-101, and BML-210 exhibited antitumor ability in orthotopic mammary tumors in mice. The epigenetic inhibitors increased antigen presentation mediated by the major histocompatibility complex class I on BC cell. Besides, BML-210 made breast tumors more susceptible to the PD-1 inhibitor. Thus, organoids can be used in ICB therapy and its combination therapy for BC. However, the material and composition of devices used in organoid cultures may affect the outcomes of these immunotherapies, including ICB therapy [87].

#### Oncolytic virotherapy

Oncolytic viruses are a class of viruses that selectively infect and damage cancer tissues without damaging normal tissues. Their use in cancers is gradually becoming a promising treatment approach [88]. However, oncolytic viruses for treating BC have not been successfully developed yet, because the traditional models adopted in previous studies failed to mimic the complex TME of human cancers sufficiently [18]. This has markedly hindered the approval of oncolytic viruses for BC treatment.

Nonetheless, organoid models show some advantages to observe the effect of oncolytic viruses in BC. Carter et al. [73] developed stable organoid models derived from BC tissues and observed the greatest oncolytic effects of oncolytic viruses that were engineered to denote a suicide gene (MeV-SCD and GLV-1h94) in the presence of the prodrug 5-fluorocytosine. They suggested that the organoid model provided a promising in vitro approach to benefit the testing and further engineering of virotherapeutic vectors in vivo. Similarly, Behrens et al. [74] also reconstructed BCO models to test the effectiveness of the oncolytic Urabe mumps virus in TNBC. They used the original oncolytic Urabe MuV clinical trial virus stock (MuV-U-Japan) that potently killed several established human BC cell lines in vitro, significantly extended the survival of nude mice with human TNBC MDA-MB-231 tumor xenografts in vivo, and exhibited significant killing activity against BC PDX cell lines grown as 3D organoids containing PDXs from patients resistant to anthracycline- and taxane-based chemotherapy. Moreover, the present study reveals the suitability of the MuV-UC virus for translation to modern clinical trials for treating patients with TNBC. However, immune cells were not included in the BC organoid models described above. More efforts are needed to incorporate immune cells into the organoid culture to improve BCOs. Once this model is successfully established, it allows a more accurate and realistic representation of the TME surrounding the tumor and the immune response of patients to oncolytic virotherapy, revealing its efficiency. It will also enable the evaluation of oncolytic viruses that have been specifically engineered to induce an immune response against tumor cells. We look forward to using such tumor-immune co-culture models to understand the potential of oncolytic virotherapy in BC treatment.

Now, we see the advantages of organoids in immunotherapy for BC. But, some important limitations of these co-culture models still exist. First, BCOs are usually derived from biopsies, which may lower the success rate of organoid modeling, and biopsy tissues often represent only a small fraction of tumor characteristics, potentially underestimating the overall complexity of BC tumors. Second, a cell culture model will miss or underestimate the genetic heterogeneity of BC tumors, especially considering BC can contain small “islands” with, e.g., cluster amplifications of Her2/neu, which are therapeutically relevant. Third, the BC–TME is complex and contains many cell types that we need to control; thus, the BC–TME usually cannot be fully replicated, even with exogenous immune components. Besides, the culture conditions of such organoids are specific, requiring many growth factors, but these growth factors may also affect other cells that are co-cultured with BCOs. A major focus of future studies should be the optimization of co-culture conditions further, considering factors such as cell growth factors and different immune cell components in the BC–TME.

## Conclusion and perspectives

Immune organoids of BC can be successfully established under certain conditions. They can potentially serve as in vitro models to evaluate sensitivity and resistance to immunotherapy, analyze new therapeutic approaches, and determine personalized immunotherapy. But we still face some challenges, such as approaches to prolong the culture time of immune organoids, solutions to vascularization, and perfusion problems. In order to fully utilize these models as immunotherapy models for BC research, it is necessary to understand their advantages and disadvantages and address the challenges we face. We believe that organoids can become a great immuno-oncology tool in BC after their successful establishment. We look forward to relative clinical trials to explore their various application values in BC research, especially for precision medicine.

## Data Availability

Data is available on request from the authors.

## Abbreviations

BC: Breast cancer
2D: Two-dimensional
3D: Three-dimensional
HER2: Antihuman epidermal growth factor receptor 2
ICB: Immune checkpoint blockade
ACT: Adoptive cell transfer
CAR: Chimeric antigen receptor
TNBC: Triple-negative breast cancer
PD-1: Programmed cell death 1
PD-L1: Programmed death ligand 1
ECM: Extracellular matrix
NK: Natural killer
TME: Tumor microenvironment
TIME: Tumor immune microenvironment
PDTX: Patient-derived tumor xenograft
PDOs: Patient-derived organoids
BCO: BC organoid
HR: Hormone receptor
ALI: Air–liquid interface
CAFs: Cancer-associated fibroblasts
BMSC: Bone marrow stromal cells
MDOTS/PDOTS: Murine- or patient-derived organotypic tumor spheroids
OoC: Organ-on-chip
TCR: T-cell receptor
TIL: Tumor-infiltrating lymphocyte
ICIs: Immune checkpoint inhibitors
iTO: Immune-enhanced tumor organoid
